# From Hyposociability to Hypersociability—The Effects of PSD-95 Deficiency on the Dysfunctional Development of Social Behavior

**DOI:** 10.3389/fnbeh.2021.618397

**Published:** 2021-01-28

**Authors:** Wen-Jun Gao, Nancy R. Mack

**Affiliations:** Department of Neurobiology and Anatomy, Drexel University College of Medicine, Philadelphia, PA, United States

**Keywords:** PSD-95, animal model, prefrontal cortex, social behavior deficit, hypersociability

## Abstract

Abnormal social behavior, including both hypo- and hypersociability, is often observed in neurodevelopmental disorders such as autism spectrum disorders. However, the mechanisms associated with these two distinct social behavior abnormalities remain unknown. Postsynaptic density protein-95 (PSD-95) is a highly abundant scaffolding protein in the excitatory synapses and an essential regulator of synaptic maturation by binding to NMDA and AMPA receptors. The *DLG4* gene encodes PSD-95, and it is a risk gene for hypersocial behavior. Interestingly, PSD-95 knockout mice exhibit hyposociability during adolescence but hypersociability in adulthood. The adolescent hyposociability is accompanied with an NMDAR hyperfunction in the medial prefrontal cortex (mPFC), an essential part of the social brain for control of sociability. The maturation of mPFC development is delayed until young adults. However, how PSD-95 deficiency affects the functional maturation of mPFC and its connection with other social brain regions remains uncharacterized. It is especially unknown how PSD-95 knockout drives the switch of social behavior from hypo- to hyper-sociability during adolescent-to-adult development. We propose an NMDAR-dependent developmental switch of hypo- to hyper-sociability. PSD-95 deficiency disrupts NMDAR-mediated synaptic connectivity of mPFC and social brain during development in an age- and pathway-specific manner. By utilizing the PSD-95 deficiency mouse, the mechanisms contributing to both hypo- and hyper-sociability can be studied in the same model. This will allow us to assess both local and long-range connectivity of mPFC and examine how they are involved in the distinct impairments in social behavior and how changes in these connections may mature over time.

Social behavior is the ability to properly communicate with others between conspecifics in both humans and animals. This function relies on coordinated processes between nodes of the “social brain” that includes the prefrontal cortex (PFC), amygdala, nucleus accumbens (NAc), anterior insula, anterior cingulate cortex, hippocampus, and temporal sulcus (Blakemore, 2008; Sandi and Haller, 2015; Porcelli et al., 2019; Kingsbury and Hong, 2020). While most have a normal social life, some experience social deficits such as hyposociability or hypersociability that are commonly seen in various neurodevelopmental and psychiatric disorders, especially autism spectrum disorders (ASD) (Haas and Reiss, 2012).

However, most of the studies focus on social impairments associated with hyposociability in adults. Recent studies highlighted hypersociability in both patients with Williams Syndrome (Porter et al., 2007; Jabbi et al., 2012) and animal models (Osborne, 2010; Barak and Feng, 2016; Barak et al., 2019; Toth, 2019). The mechanisms associated with social deficits across the spectrum remain elusive. It is especially unknown when and how hypersociability evolves during development and whether hypersociability is directly related to or derived from hyposociability that is often more frequently observed in various psychiatric disorders.

Although the precise functions of sociability-related genetic and epigenetic effects are not understood, it was proposed to converge on two neuronal processes that regulate social behavior. One is the ability of the amygdala to discriminate between fear-inducing and friendly social clues and to produce an appropriate behavioral response. The other is the dopaminergic reward/aversion system that assesses the salience of social situations and initiates approach (social reward) or avoidance (social aversion) behavior (Toth, 2019). However, a common finding focuses on the dysfunctional prefrontal cortex and its connectivity (Porter et al., 2007; Jabbi et al., 2012; Barak and Feng, 2016; Barak et al., 2019).

The dysfunctional PFC assumption is also supported by the deletion of the *DLG4* gene, which encodes postsynaptic density protein-95 (PSD-95) and is identified as a high-risk gene for hypersociability (Toth, 2019). As a member of the membrane-associated guanylate kinase (MAGUK) family (Cheng et al., 2006), PSD-95 is a core component of the PSD. Based on the quantitative mass spectroscopic assay, PSD-95 is about 6-fold more abundant than PSD-93, 8-fold more than SAP102, and 40-fold more than SAP-97 in PSDs of the adult rat forebrain (Cheng et al., 2006). Therefore, PSD-95 is the most abundant scaffolding protein in the excitatory glutamatergic synapses in the central nervous system (El-Husseini et al., 2000; Chen et al., 2011, 2015; Sheng and Kim, 2011; Fromer et al., 2014; Purcell et al., 2014). PSD-95 functions to facilitate synaptic maturation by recruiting/trafficking both N-methyl-D-aspartic acid receptors (NMDARs) and α-amino-3-hydroxy-5-methyl-4-isox-azoleproprionic acid receptors (AMPARs) to the postsynaptic membrane during neurodevelopment (Frank et al., 2016). Specifically, PDZ domains located at the C-terminus of PSD-95 bind directly with NMDAR subunits, GluN2A and GluN2B (Kornau et al., 1995), and stargazin, which adheres to AMPAR subunits (Schnell et al., 2002; Zhang et al., 2013). Therefore, it is plausible that PSD-95 dysfunction would lead to aberrant synaptic maturation and formation due to changes in NMDAR and AMPAR presence and activity. Indeed, disruption of PSD-95 expression is highly associated with synaptic dysfunction in neurodevelopmental disorders such as schizophrenia (Fromer et al., 2014) and ASD (Xing et al., 2016; Coley and Gao, 2018). A study reported that PSD-95 deficiency enhanced long-term potentiation but had limited effects on synaptic expression and function in the hippocampal CA1 pyramidal neurons (Migaud et al., 1998). However, we and others have found that the effects of PSD-95 deficiency on synaptic function are age- and brain region-specific (Béïque et al., 2006; Feyder et al., 2010; Winkler et al., 2018; Coley and Gao, 2019). Specifically, we recently reported that during adolescent development, mice with targeted deletion of the *DLG* gene (PSD-95 knockout, KO) exhibited a significant increase in NMDA-mediated excitatory synaptic currents (EPSCs) but limited effects on AMPA-EPSCs in the mPFC neurons (Coley and Gao, 2019). The question is how the synaptic changes in the mPFC induced by PSD-95 KO would affect the function of mPFC and its connections with other brain regions within and outside of the social brain circuit (Barak and Feng, 2016). *Behaviorally, adolescent PSD-95 KO mice exhibited hyposociability and impaired social novelty/memory (Coley and Gao*, *2019**), whereas adult mice displayed hypersociability despite a slight slow in locomotion (Feyder et al.*, *2010**; Winkler et al.*, *2018**)*. This phenotypic switch in sociability is fascinating. It suggests that the age-dependent and brain region-specific alterations in synaptic function induced by PSD-95 KO might be responsible for the behavioral switch from hypo- to hyper-sociability during adolescence to adulthood development.

It is worth noting that the mediodorsal thalamus (MD) is part of the limbic systems for social and emotional behaviors, but its role in the regulation of these behaviors is oddly understudied. We recently reported that the MD-mPFC pathway critically modulated social behaviors (Ferguson and Gao, 2018). Specifically, acute inhibition of the MD activity increased excitation/inhibition (E/I) balance in mPFC neurons and impaired social interaction in a three-chamber task. In adolescence, we also found that global PSD-95 KO results in an increase in evoked NMDAR-mediated excitatory postsynaptic currents (EPSCs) in the mouse prefrontal neurons and the KO mice also exhibited hyposociability. Together these findings suggest that mechanisms that increase E/I balance in both adulthood and adolescence can induce hyposociability. Given that increased inhibition of the mPFC does not increase sociability (Yizhar et al., 2011; Ferguson and Gao, 2018), we predict that a reduced NMDAR transmission, instead of an altered E/I balance, may contribute to the hypersociability. This assumption, however, remains to be tested.

Still, how does a postsynaptic scaffold protein like PSD-95 affect presynaptic function of excitatory inputs to the mPFC from the MD or other social brain regions? PSD-95 is essential in coupling the postsynaptic NMDARs to pathways that control bidirectional synaptic plasticity and learning (Migaud et al., 1998). Moreover, PSD-95 also enhances the maturation of presynaptic terminals via various mechanisms, including the enhanced size of axon terminals (El-Husseini et al., 2000), retrograde regulation of presynaptic b-neurexin via PSD-95-neuroligin interaction (Conroy et al., 2007; Futai et al., 2007), and synaptic scaling (Sun and Turrigiano, 2011). The presynaptic effect is activity- and NMDAR-dependent (Pratt et al., 2003; Südhof, 2018), and this effect could explain why the PSD-95 knockout mouse has augmented paired-pulse facilitation (Migaud et al., 1998). However, it is also possible that the presynaptic role is a secondary effect of postsynaptic maturation. Indeed, we recently reported that PSD-95 deficiency results in a significant increase in synaptic inhibition and thus a dramatic shift in the excitatory-to-inhibitory balance in the prefrontal neurons via upregulation and trafficking of neuroligin-2 and reduced GSK3β activity through tyr-216 phosphorylation (McEachern et al., 2020).

The mPFC exhibits a distinct prolonged postnatal maturation until young adulthood compared with other cortical and subcortical regions (Monaco et al., 2015). The dysregulation of mPFC development is known to contribute to the cognitive and social deficits observed in ASD (Walker et al., 2017; Coley and Gao, 2018). Especially, abnormal social approach in the Williams Syndrome is attributed to frontal lobe dysfunction (Porter et al., 2007). These findings raise a critical question of how PSD-95 deficiency during development induces prefrontal circuit and social behavior changes, i.e., the proposed aberrant short- (prefrontal intracortical) and long-range (such as MD and others) connections (Haas and Reiss, 2012), and consequently, social behavior during development. Particularly, despite the importance of PSD-95 in synaptic function and the notorious impairment of synaptic dysconnectivity seen in both SCZ and ASD (Tsatsanis et al., 2003; Nair et al., 2013; Buchmann et al., 2014), it remains unknown whether PSD-95 deficiency would also affect synaptic function and connectivity in the mPFC. NMDA receptors are essential for the social brain (Ferri et al., 2020) and social behavior development in rodents (Zoicas and Kornhuber, 2019). Activation of NMDARs regulates sociability (Burket et al., 2015), and NMDAR dysfunction is a prominent pathophysiological feature in ASD (Won et al., 2012; Coley and Gao, 2018; Chung et al., 2019). A laminar- and pathway-specific expression and development of NMDARs in different mPFC connections, as reported in the MD-mPFC pathway (Miller et al., 2017), are likely the targets of PSD-95 deletion. In particular, normally there are GluN2B-to-GluN2A subunit switches in many brain regions during postnatal development, but this process is prolonged in the mPFC (Monaco et al., 2015). This natural NMDAR2 subunit switch represents the maturation of development that is related to associative learning (Dumas, 2005). In contrast, PSD-95 KO resulted in an increase in GluN1 and GluN2B expression in the mPFC, and the predominant NMDARs were thus dominated with the expression of GluN2B-NMDARs that have a higher conductance and thereby increased NMDA-EPSCs and NMDA/AMPA ratio (Coley and Gao, 2019). Such a change in NMDAR composition in PSD-95 KO neurons is a reversal to an immature state of the synapse and most likely is the root cause behind the delayed maturation of the prefrontal circuitry and its aberrant connectivity, as we proposed here.

Therefore, utilizing PSD-95 deficient mouse models (heterozygous PSD-95^+/−^ and homozygous PSD-95^−/−^) to explore the effects on both local excitatory and long-range connectivity in the mPFC and their roles in social behavior during development will provide a better understanding of how social behavior evolves from hypo- to hyper-sociability. To our knowledge, most developmental disorders exhibit either hypo- or hyper-sociability. Even in animal models for ASD, social behavior is often tested in adulthood, leaving the question of developmental alteration of social behavior uncharacterized. Therefore, the developmental phenotypic switch is unique and PSD-95 deficient mouse model offers a specific template to studying mechanisms that underly both hypo and hyper sociability in one model. This can be achieved by using a combination of physiological and optogenetic techniques, as well as behavioral tasks, to test this hypothesis.

There are also several remaining questions to further understand regarding the role of PSD-95 in regulating synaptic function in the PFC. First, PSD-95 family MAGUKs are essential for anchoring AMPA and NMDA receptor complexes at the postsynaptic density (Chen et al., 2015). However, how MAGUKs underlie synaptic strength is not well understood. A recent study explored the structural and functional roles of MAGUKs at hippocampal excitatory synapses by simultaneously knocking down PSD-95, PSD-93, and SAP102 and combining electrophysiology and transmission electron microscopic tomography imaging to analyze the resulting changes (Chen et al., 2015). Acute MAGUK knockdown significantly reduces synaptic transmission mediated by AMPARs and NMDARs but leads to a significant rise in the number of silent synapses (Chen et al., 2015). This change in silent synapses, which contains postsynaptic membrane with NMDARs but no AMPARs, is consistent with the increased GluN1 and GluN2B expression and NMDAR-mediated currents in PSD-95 KO mice (Coley and Gao, 2019). Moreover, NMDA receptors are selectively partitioned into complexes and tripartite GluN2B, PSD-93, and PSD-95 supercomplexes during synaptic maturation (Frank et al., 2016). Specifically, NMDAR supercomplexes are assembled late in postnatal development and are triggered by synapse maturation involving epigenetic and activity-dependent mechanisms (Frank et al., 2016). However, the role of these supercomplexes, how PSD-95 KO affects these supercomplexes, and their connection to aberrant social behavior remain to be determined.

Nevertheless, because PSD-MAGUKs share a common domain structure, including three PDZ (PDZ1/2/3) domains in their N-terminus, ligand binding-deficient PSD-95 knockin (KI) mice showed decreased accumulation of mutant PSD-95, PSD-93, and AMPA receptor subunits in the PSD fraction of the hippocampus (Nagura et al., 2012). As expected, PSD-95 has an age- and subregion-dependent role in regulating synaptic function and plasticity. In the hippocampal CA1 region of young KI mice, basal synaptic efficacy was reduced, and LTP was enhanced with intact LTD. In contrast, in adult KI mice, there was no significant change in the magnitude of LTP in CA1, but robustly enhanced LTP was induced at the medial perforant path-dentate gyrus synapses (Nagura et al., 2012). Adult KI mice showed markedly abnormal anxiety-like behavior, impaired spatial reference and working memory, and impaired remote memory and pattern separation in the fear conditioning test. Thus, PSD-95 controls the synaptic clustering of PSD-MAGUKs and glutamatergic receptors, which is essential in regulating hippocampal synaptic transmission, plasticity, and hippocampus-dependent behavior (Nagura et al., 2012). This is in agreement with a previous report that social isolation produced anxiety-like behaviors and changed PSD-95 levels in a brain region-specific manner, i.e., PSD-95 levels were elevated in the hippocampus and amygdala but reduced in the frontal cortex after social isolation (Zhang et al., 2012).

An arguable notion is that a global KO instead of region-specific deletion might not be ideal to understand the circuit dysfunction, and analysis of afferent-specific pathways in the context of such global PSD-95 deficiency may not seem to be useful. However, a recent study indicates that conditional SAP-97 KO in the hippocampus exhibited only subtle male-specific cognitive deficit and female-specific motor deficit, while other behaviors were mostly unaffected, including social behavior (Gupta et al., 2018). It is likely that a global connectivity problem instead of change in a single region is required for our proposed social behavioral changes in PSD-95 KO mice. We predict that PSD-95 KO induces increased silent synapses in prefrontal neurons during the unique prolonged postnatal development (Coley and Gao, 2019). This reversal of cortical maturation results in an aberrant prefrontal circuit and long-range disconnection with other brain regions, which in turn, induce behavioral changes in an age-dependent manner.

However, this assumption remains to be tested and/or challenged because previous studies reported different effects of loss of PSD proteins on synaptic AMPARs and NMDARs and synaptic plasticity in different brain regions (Elias et al., 2006; Carlisle et al., 2008; Krüger et al., 2013). Specifically, PSD-95 and PSD-93 have opposing roles in the maturation of silent synapses in the visual cortex, hippocampus, and PFC (Favaro et al., 2018). While PSD-95 promotes the maturation of silent synapses, PSD-93 acts as a brake and slows the process (Favaro et al., 2018). Because of this differential regulation, the fraction of silent synapses declines faster with a different developmental trejactory, and a critical period of visual cortex development closes too early in mice lacking PSD-93. Consequently, visual acuity appears not to be affected in mice that lack either PSD-93 or PSD-95, but is severely reduced in mice with deficiency of both proteins. Proper orientation discrimination in adult mice also requires both PSD-93 and PSD-95 (Favaro et al., 2018). These results indicate that both PSD-95 and PSD-93 are mutually dependent and required for balanced functions that are crucial for normal development of synapses and optimal brain function. Indeed, Levy and Nicoll demonstrated an age-dependent requirement of MAGUKs in controlling synaptic strength that corresponds to a period of heightened plasticity in pre-adolescent rats and shows the importance of temporally controlled manipulations (Levy and Nicoll, 2017).

Another question is whether the shift in behavioral phenotypes with age in the PSD-95 KO mice is attributable to the distinct downstream signaling pathways and/or the upstream NMDAR-mediated transmission defect. This is indeed an interesting question that deserves additional investigations. Further, constitutive KO of PSD-95 will not only disrupt NMDAR function, but such deficiency even in heterozygotes will also likely induce homeostatic changes in synapse function (Keck et al., 2017). These changes could be maladaptive and result in synaptic dysfunction and circuit aberration that are associated with other family members of the MAGUKs. Indeed, there is robust hypersocial behavior in the dyadic interaction test in both adult PSD-95^+/−^ males and females, suggesting hypersocial behavior and biological redundancy in mice with reduced expression of PSD-95 or PSD-93 (Winkler et al., 2018). Additionally, PSD-93 homozygous (but not heterozygous) KO mice displayed similar prominent hypersocial behavior comparable to that observed in PSD-95^+/−^ mice, despite a more severe motor phenotype. There was also increased PSD-93 protein expression in hippocampal synapse of PSD-95^+/−^ mice, whereas the changes in the mPFC and other regions were not examined (Winkler et al., 2018). Consistently, DLG2 (also known as PSD-93 or chapsyn-110) deficient in mice also led to reduced sociability and increased repetitive behavior accompanied by aberrant synaptic transmission in the dorsal striatum (Yoo et al., 2020). These data further suggest that both PSD-95 and PSD-93 are involved in processing social stimuli and social behavior control, indicating their functional redundancy (Winkler et al., 2018). PSD-95 as a key molecule in synapses plays a critical role in regulating the subunit composition of NMDARs and the level of AMPARs and its activity-dependent change at synaptic sites via interactions with PDZ domains (Migaud et al., 1998; Béïque et al., 2006; Elias et al., 2006, 2008; Carlisle et al., 2008; Sun and Turrigiano, 2011; Xu, 2011). However, due to functional redundancy among PSD-MAGUKs and their multiple protein-interacting domain structure, the specific roles of individual PSD-MAGUKs during development *in vivo* remains unclear. Future studies will focus on how these diversities are achieved and to what extent PSD-MAGUKs and their components, individually or jointly, contribute to orchestrating the signaling cascades and synaptic development for the various types of synaptic plasticity at different glutamatergic synapses (Xu, 2011).

With all these considerations, what we proposed here is to study an age-dependent and circuit-specific social behavior switch from hypo- to hyper-sociability using the PSD-95 mouse model. This perspective plan is the first attempt to characterize the effects of PSD-95 deficiency specifically on mPFC synaptic function by examining glutamatergic transmission from thalamocortical projections in response to PSD-95 deficiency compared to afferents from other social brain regions such as the hippocampus and amygdala. This approach will isolate afferent specific fibers that are critical for the maturation of the mPFC. While previous studies showed that PSD-95 knockdown altered NMDAR and AMPAR expression and function in cultured hippocampal neurons, oddly, the effects of PSD-95 on both local and long-range connectivity of prefrontal neurons have never been characterized. Using the unique PSD-95^+/−^ and PSD-95^−/−^ mice combined with morphological, physiological, and optogenetic techniques, this study will address an intriguing hypothesis of how NMDAR-mediated synaptic function and connection in the mPFC is associated with the sociability switch from hypo- to hyper-social status during the adolescent development. These results will undoubtedly provide novel insights into the understanding of how PSD-95 deficiency affects PFC-associated regulation of social behavior across development and its potential implications in neuropsychiatric disorders such as the ASD.

## Author Contributions

W-JG and NRM wrote the paper. All authors contributed to the article and approved the submitted version.

## Conflict of Interest

The authors declare that the research was conducted in the absence of any commercial or financial relationships that could be construed as a potential conflict of interest.
